# Effect of Strontium Substitution on the Physicochemical Properties and Bone Regeneration Potential of 3D Printed Calcium Silicate Scaffolds

**DOI:** 10.3390/ijms20112729

**Published:** 2019-06-03

**Authors:** Yung-Cheng Chiu, Ming-You Shie, Yen-Hong Lin, Alvin Kai-Xing Lee, Yi-Wen Chen

**Affiliations:** 1 School of Medicine, China Medical University, Taichung 40447, Taiwan; ycchiu@me.com (Y.-C.C.); leekaixingalvin@gmail.com (A.K.-X.L.); 2 Department of Orthopedic Surgery, China Medical University Hospital, Taichung 40447, Taiwan; 3 School of Dentistry, China Medical University, Taichung 40447, Taiwan; eric@mail.cmu.edu.tw; 4 3D Printing Medical Research Center, China Medical University Hospital, Taichung 40447, Taiwan; roger.lin0204@gmail.com; 5 Department of Bioinformatics and Medical Engineering, Asia University, Taichung 40447, Taiwan; 6 The Ph.D. Program for Medical Engineering and Rehabilitation Science, China Medical University, Taichung 40447, Taiwan; 7 Graduate Institute of Biomedical Sciences, China Medical University, Taichung 40447, Taiwan; 8 3D Printing Medical Research Institute, Asia University, Taichung 40447, Taiwan

**Keywords:** calcium silicate, strontium, 3D printing, scaffold, osteogenesis, osteoclastogenesis

## Abstract

In this study, we synthesized strontium-contained calcium silicate (SrCS) powder and fabricated SrCS scaffolds with controlled precise structures using 3D printing techniques. SrCS scaffolds were shown to possess increased mechanical properties as compared to calcium silicate (CS) scaffolds. Our results showed that SrCS scaffolds had uniform interconnected macropores (~500 µm) with a compressive strength 2-times higher than that of CS scaffolds. The biological behaviors of SrCS scaffolds were assessed using the following characteristics: apatite-precipitating ability, cytocompatibility, proliferation, and osteogenic differentiation of human mesenchymal stem cells (MSCs). With CS scaffolds as controls, our results indicated that SrCS scaffolds demonstrated good apatite-forming bioactivity with sustained release of Si and Sr ions. The in vitro tests demonstrated that SrCS scaffolds possessed excellent biocompatibility which in turn stimulated adhesion, proliferation, and differentiation of MSCs. In addition, the SrCS scaffolds were able to enhance MSCs synthesis of osteoprotegerin (OPG) and suppress macrophage colony-stimulating factor (M-CSF) thus disrupting normal bone homeostasis which led to enhanced bone formation over bone resorption. Implanted SrCS scaffolds were able to promote new blood vessel growth and new bone regeneration within 4 weeks after implantation in critical-sized rabbit femur defects. Therefore, it was shown that 3D printed SrCS scaffolds with specific controllable structures can be fabricated and SrCS scaffolds had enhanced mechanical property and osteogenesis behavior which makes it a suitable potential candidate for bone regeneration.

## 1. Introduction

Trauma, tumors, and diseases can lead to bone defects and the problem of large bone defects had been troubling surgeons as there are currently no appropriate treatment solutions for large bone defects [1]. Furthermore, an increasing aging population has also contributed to a greater number of cases of bone defects due to increased risks of diseases, inflammation, and tumors. Therefore, there has been a surge in bone substitute research in the orthopedics field to attempt to find suitable bone substitutes for bone defects [2]. The emergence and development of regenerative medicine and 3D printing have made bone tissue engineering a potential solution for bone substitutes [3,4,5]. In recent years, several studies have demonstrated that 3D-printed scaffolds support bone regeneration and factors—such as porosity, pore size, pore shape, pore distribution, and pore interconnectivity of the scaffold—which play a part in determining bone regenerative effects [6,7,8]. Bones are highly vascularized and homeostasis of bone metabolism is highly dependent on the tight knit interactions between blood vessels, bone cells, and numerous signaling molecules [9]. The vasculatures provide an avenue for provision of essential nutrients, growth factors, cytokines, and chemokines and also regulate metabolic waste removal. An important fact to note is that both angiogenesis and osteogenesis must exist simultaneously for proper and efficient bone healing [10].

Therefore, there is a slight paradigm shift in the current trend of bone grafting materials. Researchers are now attempting to insert trace metallic ions into materials as such a method has lower regulatory restrictions and risks as compared to osteoinductive methods. It was reported that incorporation of certain specific metallic ions into bone substitute biomaterials may enhance bioactivity, biocompatibility, mechanical, and antimicrobial properties [11,12,13,14]. A commonly used material for bone tissue engineering, known as calcium silicate (CS)-based ceramics, is known for its potential in guiding new bone growth and regeneration and has a higher osteoconductivity, osteoinductivity, and biocompatibility than other calcium phosphate-based materials [15]. CS has ideal surface properties that allow efficient cellular adhesion and CS is also known to be able to have a sustained release of ions into surrounding fluids that are heavily involved in regulating osteogenesis. It is widely reported that the released Si ion has a potential in positively influencing cellular proliferation and subsequent osteogenesis [16]. Besides, studies have also shown that silicate-based materials could directly or indirectly promote the expression of angiogenic factors. In addition, the degrading CS scaffold releases Ca ion which would react with PO_4_^−3^ in the biological fluid followed by mineralizing on the pre-formed SiO_2_ surface of the scaffold [17]. Such mineralization would result in the formation of a hydroxyapatite coating which was reported to aid in subsequent tissue-scaffold binding [18].

Previously, we had developed CS-based materials and initial results had shown that CS-based materials were able to enhance expression of osteo- and angiogenic factors in various primary cell lines and ultimately stimulate the proliferation and differentiation of osteogenic cells [19,20]. Thus, initial results indicated that CS-based ceramics have potential for future clinical applications. A novel method of inducing Sr into CS may be used to further enhance the biological and mechanical properties of CS-based materials which could led to enhanced bone formation. Sr is an indispensable trace element in the human body and 99.1% of absorbed Sr is found to be deposited in teeth and human bones, particularly new formed bones [21]. Sr and Ca are known to be part of the alkali metals; thus, they tend to share very similar kinetic characteristics in vivo and Sr has since attracted the attention of numerous researchers [22]. Sr is highly related to the regulation of bone metabolism and many studies have demonstrated that Sr possesses this “dual regulation” which is known to stimulate osteoblasts to secrete new bone matrix while simultaneously inhibiting osteoclast activity and reducing bone resorption. Inhibiting the receptor activator of nuclear factor κ-B ligand (RANKL)/receptor activator of nuclear factor κ-B (RANK)/osteoprotegerin (OPG) signaling pathway would inhibit the process of bone resorption for osteoclasts [23]. Both RANK and OPG signaling molecules can be expressed by osteoblasts and their precursor cells. Binding of RANK receptor on the surface of osteoclast precursor cells would lead to signal transduction and osteoclast differentiation to mature osteoclasts and therefore break down mineralized bone tissue. In contrast, OPG is a decoy receptor that and is regulated by Sr ions secreted by osteoblast [24]. Activation of this decoy receptor would inhibit osteoclast differentiation and also bone resorption. In addition, Sr can also mediate the calcium-dependent cellular signaling pathway through the calcium-sensing receptor (CaSR) which is a common physiological receptor for osteoclasts, osteoblasts, and bone cells which would lead to further activation of the mitogen-mediated protein kinase (MAPK) modules [25]. As mentioned, Sr and Ca are closely related and therefore this could be used to explain why other di- or trivalent cations could also promote bone remodeling at a certain dose.

There are many factors influencing cellular behaviors in engineered scaffolds and usually such factors are multi-faceted. Scaffolds for bone regeneration should have good levels of bioactivity and biodegradability and at the same time have suitable mechanical properties. Other than the materials or components of scaffolds, design also plays a huge role in regulating the above-mentioned factors. Traditional manufacturing methods often fail to achieve desired characteristics such as specific pore sizes, pore structures, porosity, and inter-connections of pores, thus making it unsuitable for cell growth and clinical applications. However, 3D printing technology has since overcome such problems and disadvantages. Sr has been used to modify numerous synthetic materials such as chitosan, collagen, and montmorillonite [26]. To our knowledge, No et al. conducted the first ever attempted study to modify CS with Sr [27]. Our studies had previously proved the efficacy and effects of CS. In this study, we would synthesize strontium-contained calcium silicate (SrCS) powder and the main aim of this study is to evaluate its potential in future bone engineering clinical applications by looking into the factors behind enhanced bone regeneration and to assess its mineralization capabilities via in vitro and in vivo studies.

## 2. Results and Discussion

### 2.1. Characterization of SrCS Scaffolds

**Figure 1:**
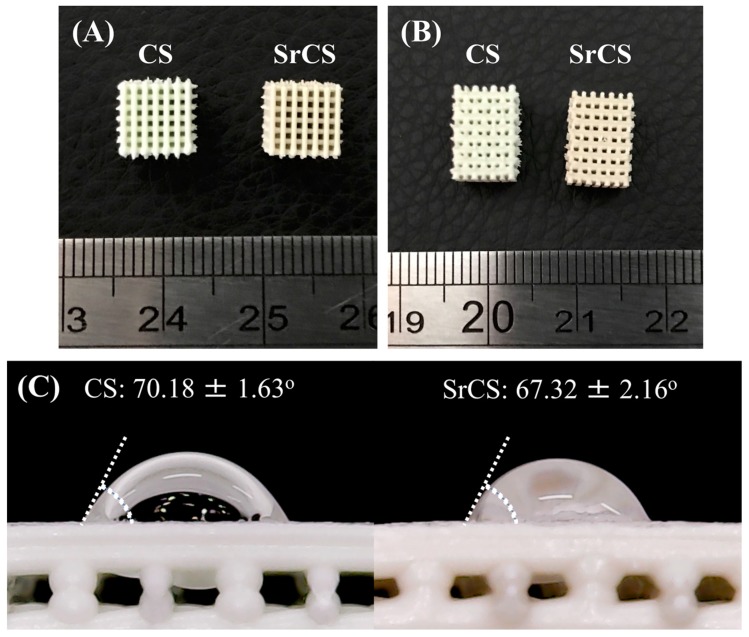
Top view (**A**) and lateral view (**B**) of the printed CS and SrCS scaffolds. Water contact angle of (**C**) CS sand SrCS scaffold. Data presented as mean ± SEM, *n* = 6 for each group.

Printed CS scaffolds were mint-green in color and printed SrCS scaffolds were brown in color as seen in Figure 1. Photo images of both scaffolds showed that the struts and surfaces were noticeably smooth and continuous thus indicating that the addition of Sr do not affect printing and scaffold quality. A square shaped scaffold of 6.5 × 6.5 × 10 mm was fabricated with 500 μm pores. As reported by others, the geometry of scaffolds were critical in significantly enhancing cellular response and rate of bone tissue generation and current evidence has shown that a minimal pore size of a few hundred μm were necessary for successful bone regeneration [28]. From the images, it can be clearly seen that the pores were obvious and constant and both CS and SrCS material can be printed and stacked to form scaffolds. The water contact angle on the scaffolds was measured using a sessile drop technique. There were no significant differences (*p* > 0.05) between the water contact angle of CS scaffolds (70.17 ± 1.63°) and SrCS scaffold (67.32 ± 2.16°). Our results were consistent with studies made by others which further confirmed that addition of Sr made biomaterials hydrophilic [29]. Similarly, it had been stated that hydrophilicity led to enhanced osteoconductivity and improve subsequent bone tissue regeneration [30]. Therefore, the presence of Sr was shown to hydrophilize 3D printed porous CS scaffolds which can improve cell–biomaterial interactions and enhance diffusion of cell culture medium and nutrient transfer into the scaffold, thus leading to increased tissue growth and regeneration [31].

**Figure 2:**
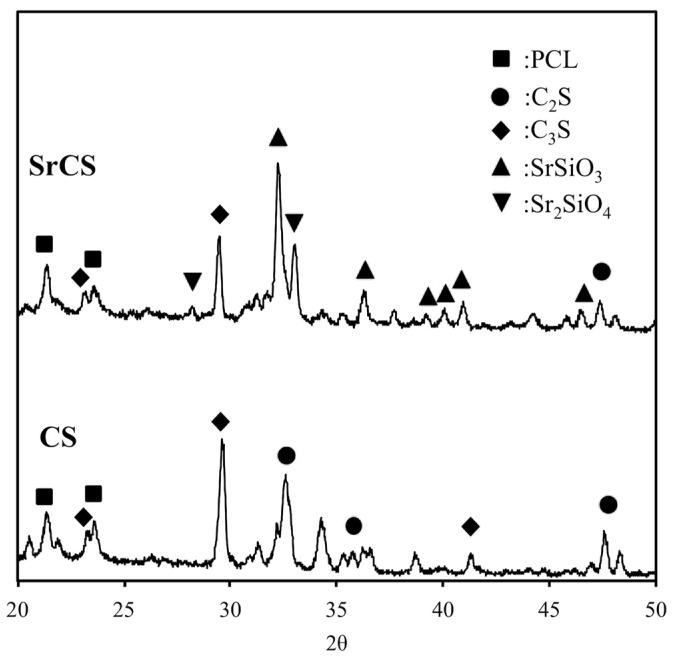
X-ray diffraction patterns of CS and SrCS scaffolds.

The X-ray diffractometry analysis for SrCS showed diffraction peaks at 29.4°, 33.5°, 33.8°, and various smaller peaks between 38.7° to 42.8° (Figure 2). Although there was a decrease in the intensity of the Ca_2_SiO_4_ peak at the 29.4° mark, diffraction of Ca_2_SiO_4_ could still be noted along the analysis result thus strongly indicating that CS was still present in the compound structure and modification with Sr did not alter the original structural properties of CS. The reduction of Ca concentration was consistent with results made by others in the sense that Sr modification resulted in microstructural reordering of CS and thus affecting crystallinity. Furthermore, presence of Sr peak at the 33.5° mark, together with the presence of other SrSiO_3_ and Sr_2_SiO_4_ diffraction peaks confirmed the successful incorporation of Sr into CS. In addition, it was worthy to note that an ideal scaffold must be able to release Sr ion in a sustained manner in order to achieve and maintain therapeutic effect and yet, at the same time, this release rate must be controllable to avoid detrimental effects [16]. It was reported that a high strontium concentration induces defective bone mineralization whilst a low level was able to induce effective bone formation. Thus, this study aimed to conduct further testing to assess for physicochemical properties and osteogenic capabilities of SrCS as compared to CS scaffolds.

**Figure 3:**
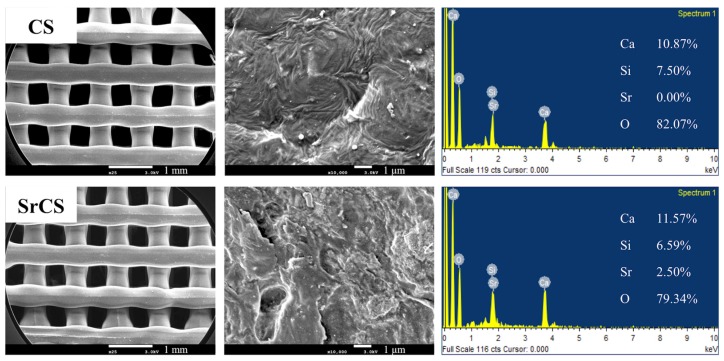
Surface SEM images of the CS and SrCS scaffolds and evolution of different ions by energy dispersive spectroscopy. The scale bar is 1 mm (low magnification) and 1 µm (high magnification).

In addition, SEM was applied to analyze for the morphology and microstructures of the scaffolds as seen in Figure 3. As noted with the macroscopic view in Figure 1, both the CS and SrCS had smooth and contiguous struts thus indicating that the addition of Sr did not alter printing capabilities of CS. In addition, in both scaffolds, the micropores were shown to remain interconnected and were clearly visible with a pore size of about 500 μm. As mentioned, this characteristic is ideal as it has the potential to induce new bone regeneration and growth. EDS analysis from Figure 3 shown that SrCS contained higher concentrations of Sr and Ca as compared to the CS scaffolds. There was a decrease in Si and O concentrations as compared to the CS scaffolds. However, this test and XRD results above serve to inform us that the original compositions of the CS components were still present even after addition of Sr.

**Figure 4:**
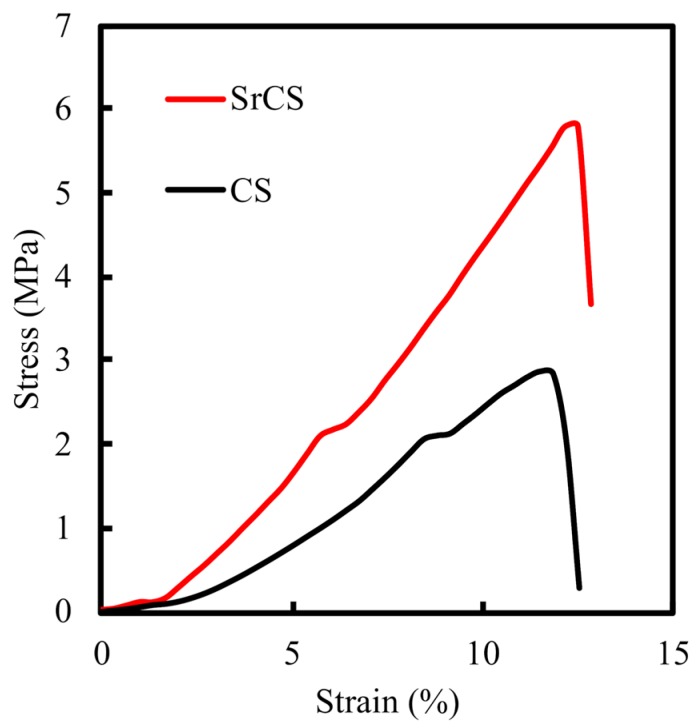
Compression stress–strain curves of 3D-printed CS and SrCS scaffolds.

Representative stress–strain curves of both SrCS and CS scaffolds were shown in Figure 4. From Figure 4, it was clearly indicated that SrCS had significantly higher mechanical properties as compared to neat CS scaffolds with approximately 5.9 MPa for the SrCS scaffold and 2.6 MPa for the neat CS scaffolds. There was a 2-fold increase in mechanical properties which is an ideal and important characteristic to possess especially for bone tissue regeneration [8]. The addition of Sr provided higher numbers of functional groups that allowed more ions to be bonded and thus increasing the mechanical properties. Different bones in our body were subjected to different mechanical stress according to their function and age and bones generally withstand a pressure of 80–120 MPa. A recent study with modified porous nano-hydroxyapatite/chitosan/tripolyphosphate for fabricating bone scaffolds showed an enhance compressive strength of 3.93 MPa which was roughly computed to be 79.98 MPa in accordance with the ASTM-C39-05 criteria [32]. Therefore, this initial result showed that addition of Sr improved tensile strength of printed scaffolds thus making them more suitable for subsequent implantations [33]. These improved mechanical properties allow more room for surgical handlings and procedures which could lead to shortened surgical duration and better healing [34].

### 2.2. Bioactivity of SrCS Scaffolds

**Figure 5:**
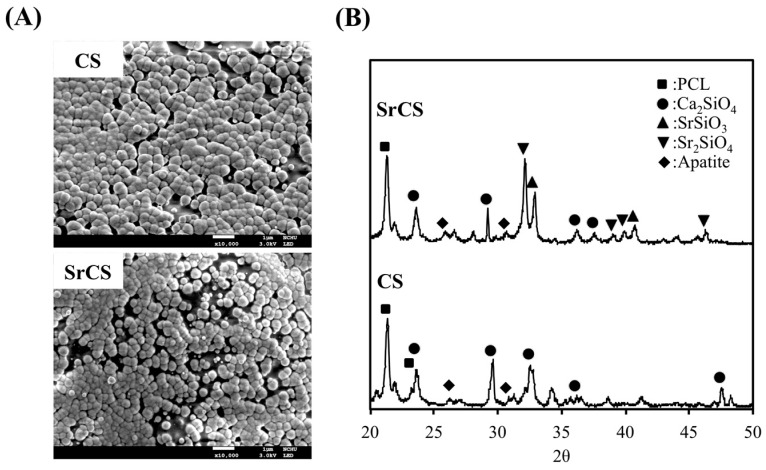
(**A**) Surface SEM images and (**B**) X-ray diffraction patterns of CS and SrCS scaffold after immersed in SBF for 3 days. Scale bar: 1 µm.

**Figure 6:**
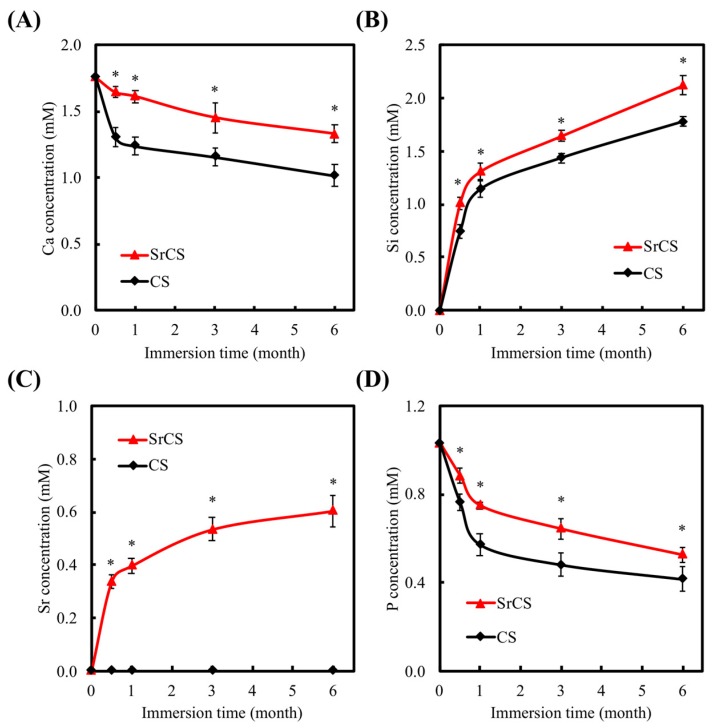
(**A**) Ca, (**B**) Si, (**C**) Sr, and (**D**) P ion concentrations in SBF after immersion for different durations. Data presented as mean ± SEM, *n* = 6 for each group. “*” indicates a significant difference (*p* < 0.05) when compared to CS scaffold.

The results of post immersion apatite precipitation were also shown in Figure 5. The formation of bone like apatite was reported to be significant in predicting subsequent bone forming capabilities [35]. A layer of spherical materials of apatite was precipitated on the scaffold surface after immersion in SBF for 3 days due to the Ca and Si ions dissolved from the scaffold, indicating a fast apatite- precipitated ability for both CS and SrCS scaffolds. In addition, a similar thickness of apatite-precipitated layer and a spherical morphology can be examined for CS and SrCS scaffold that suggested the ability to form apatite on CS scaffolds have not been affected by the substitution of Sr ion for Ca ion [36]. Figure 6 showed the values of Ca, Si, Sr, and P ion concentrations after being immersed in SBF for different durations. For SrCS, the concentrations of all Ca, Si, Sr, and P ions in SBF were significantly higher (*p* < 0.05) than CS scaffolds at all time points. The ion concentrations of SrCS in SBF were approximately at 1.45 mM, 2.02 mM, 0.60 mM, and 0.55 mM for Ca, Si, Sr, and P ions respectively as compared to 1.05 mM, 1.60 mM, 0.00 mM, and 0.42 mM respectively for CS scaffolds. Si ions were rapidly released in the first 1 to 2 months and dropped to a gradual release from the second month onwards. In human body, the Sr concentration of skeleton tissue is about 3.5 mol% of Ca content [37]. Therefore, the Sr ion concentration released from SrCS scaffold is not higher than the Sr concentration found in human bone. For Sr, it was to be expected that only SrCS release Sr ions and Sr ions similarly were released rapidly during the first month and gradually declined to a steady immersion from the first month onwards. In contrast, the Ca and P ions were gradually declined over the months of immersion. Similarly, other studies with Sr had shown similar burst release profiles of Sr ions and that incorporation of Sr ions had significant pro-osteogenic potential such as inducing higher amount of hydroxyapatite layer formation [38]. In similar studies, the presence of Sr was not only found to stimulate pre-osteoblastic cells, Sr was also found to have anti-inflammatory responses which were a critical component to have as the main problem of nano- and micro-sized particles application is in its immune system activation [39]. In addition, numerous reports had indicated that Si, being a trace element, had a huge role to play in enhancing bone growth and Si had been widely applied to biomaterials due to its osteogenic potential. Furthermore, it was reported that a Si ion concentration of 0.0625 mM could counteract the effect of the WNT inhibitor, thus reducing inflammatory responses [23]. Therefore, our initial results indicated SrCS had a potential role to play in improving bioactivity of CS scaffolds.

### 2.3. Biocompatibility

**Figure 7:**
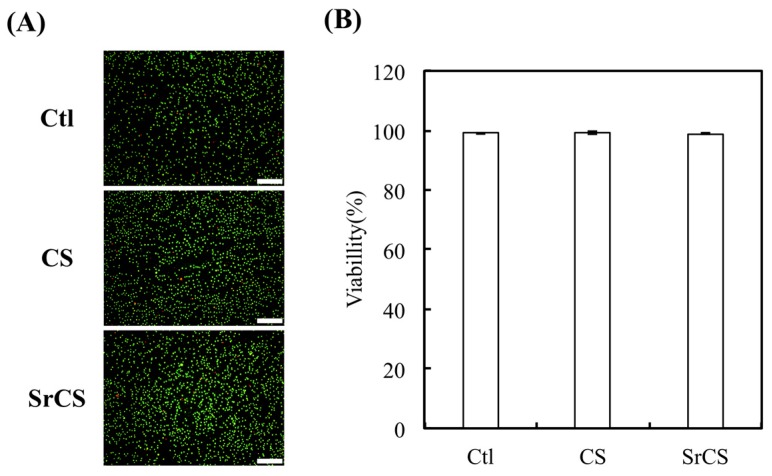
(**A**) The image and (**B**) quantification of live/dead assay results of L929 cultured in CS or SrCS extracts after 24 h cultivation. Data presented as mean ± SEM, *n* = 6 for each group. Scale bar: 400 µm. Ctl: the cultured plate.

Indirect cytotoxicity testing using live/dead assays is an important method for considering the cytotoxicity, especially so for evaluating the cytotoxicity of biomaterials that the products released from the scaffolds that modified by ISO 10993-12 [40]. First, we confirmed cell cytotoxicity of the extract of CS or SrCS by live/dead staining and fluorescence microscopy. The fluorescent images revealed considerable L929 viability (green) and there were few cells with red in three groups (Figure 7A). Then, the quantitative results were showed as the percentage of viable L929 cells in the presence of different extracts of CS and SrCS as compared to Ctl (the cultured plate), which represent the L929 cells cultured with DMEM (Figure 7B). The cytotoxic assay demonstrated that CS and SrCS scaffolds were non-toxic to L929 [41]. There was no significant difference of the cell viability between Ctl, CS, and SrCS, which exhibits the CS and SrCS scaffolds were biocompatibility.

### 2.4. Cell Proliferation and Morphology

**Figure 8:**
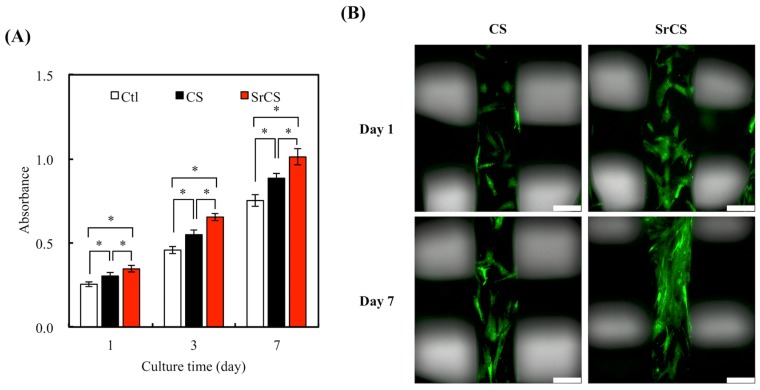
(**A**) Proliferation of MSCs on CS and SrCS scaffolds for different durations and (**B**) F-actin immunofluorescence stains. Data presented as mean ± SEM, *n* = 6 for each group. “*” indicates a significant difference (*p* < 0.05) between two groups. “Ctl” represented cells that grew in empty wells without any scaffolds. Scale bar: 250 µm.

Cell proliferation assays for 1, 3, and 7 days were performed using PrestoBlue cell viability reagents and the absorbance readings are indicated in Figure 8A. The Ctl used in this study is cell culture on culture dishes without scaffolds. It can be seen that the addition of Sr into CS enhanced the proliferation rate of MSCs on day 1 as compared to neat CS. Meanwhile, CS also displayed a significant amount of increase in cell proliferation on day 1 as compared to Ctl (*p* < 0.05). After which, cells proliferated at a fixed rate after day 1 with higher cellular numbers seen on days 3 and 7. These results clearly indicated that the presence of Sr promoted MSCs proliferation and it could be hypothesized that the hydrophilic nature of the SrCS scaffolds was favorable for cellular adhesion and attachment, which led to enhanced proliferation [42]. Kendler indicated the Sr ion-contained culture medium affected the osteoblast-like cells in increased extracellular matrix protein synthesis and enhanced the differentiation behavior of osteoblast [43]. Furthermore, the increased proliferation of MSCs could be related to the release kinetics of Sr and Si as mentioned above. Reports had stated that the increased availability of additional surface ligands from Sr could play a role in allowing extracellular matrix adsorption thus leading to increased proliferation. However, the exact mechanism of how Sr enhances cellular proliferation remains to be explored [41]. Figure 8B showed the F-actin immunofluorescence stains to visualize for cellular adhesion and spreading. At day 1, MSCs cultured on SrCS had been flat with a well-defined morphology, whilst rounded morphologies of some cells on the CS were observed that strongly indicating that cells were not being properly adhered onto the CS scaffolds [30]. The degree of cellular adhesion was reported to be strongly indicative of further downstream cellular behaviors such as cell proliferation and differentiation. In addition, it was similarly obvious that there were more cells on the SrCS scaffolds as compared to CS scaffolds [41]. Therefore, these results indicated that SrCS scaffolds were non-toxic to cells and had properties that were able to support cell adhesion and increased cell proliferation for long term cultures.

### 2.5. MAPK Pathway

**Figure 9:**
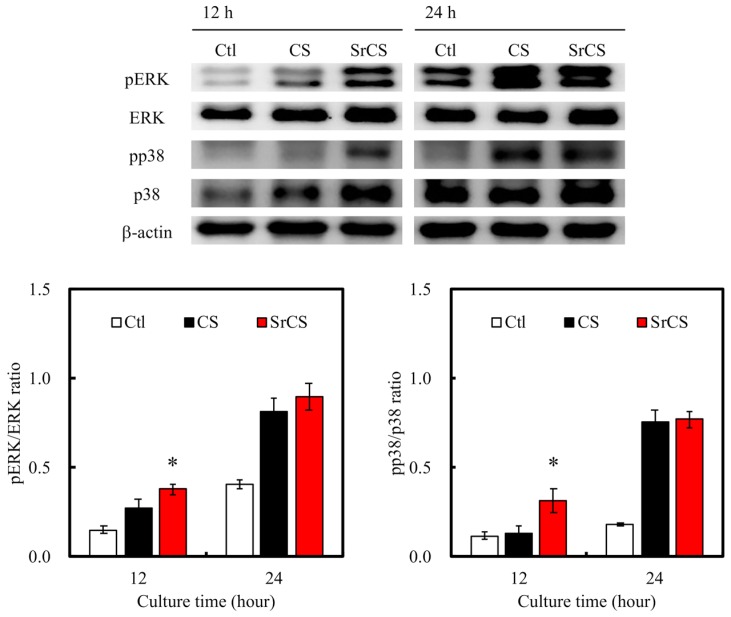
Western blotting of pERK, ERK, pp38 and p38 protein expressions of MSCs cultured on the various specimens for 12 and 24 h. “*” indicates a significant difference (*p* < 0.05) when compared to CS scaffold. Data presented as mean ± SEM, *n* = 3 for each group. “Ctl” represented cells that grew in empty wells without any scaffolds.

To further quantify for the level of cellular adhesion and proliferation, the levels of several cell signaling proteins were assessed using western blotting (Figure 9). RNA-dependent protein kinase (PKR)-like ER kinase (pERK), extracellular signal-regulated protein kinases (ERK), p38 mitogen-activated protein kinases, and β-actin were measured. MSCs cultured on SrCS had a higher expression levels of pERK, pp38, and p38 after 12 and 24 h of culture. There were no notable differences in expression of ERK and β-actin. In addition, quantitative data for pERK/ERK ratio showed that there was a significant difference between SrCS and CS at 12 h but had no significant differences at 24 h. The results were similar for pp38/p38 ratio. Such protein kinases are critical components involved in the signaling network that allow cells to carry out specific downstream functions such as differentiation or proliferation. A number of protein kinases were extensively studied in the past decades and a family of protein kinase, termed the mitogen-activated protein kinases (MAPK), were found to have a huge role to play in many diseases such as cancer, obesity, cardiovascular diseases, and pulmonary diseases. Extracellular stimuli trigger off a series of chain reactions by first interacting with a receptor tyrosine kinases and G-protein coupled receptor which led to downstream activation of MAPK. Once activated, MAPK would set off to phosphorylate a number of other substrates and regulate cellular behaviors. pERK, ERK, pp38, and p38 have been identified as members of this MAPK, therefore, they are often used as a significant biomarker for levels of cellular adhesion and proliferation [44]. As seen in Figure 9, increased pERK, pp38, and p38 expression in the SrCS group was closely correlated to the increased cellular adhesion and proliferation data as stated above. In addition, such results were consistent with reports made by others and thus indicated that SrCS could be used as a potential biomaterial for bone tissue regeneration [45].

### 2.6. Osteogenic Differentiation Ability

**Figure 10:**
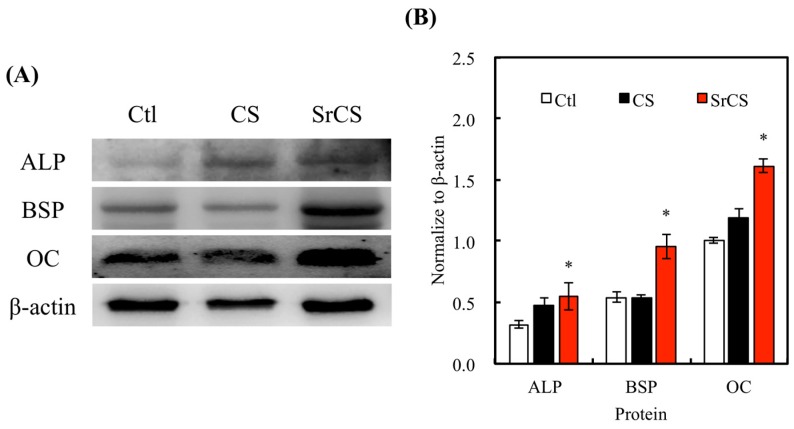
(**A**) The expression levels of osteogenic-related protein ALP, BSP, and OC protein in MSCs cultured with various scaffolds for 7 days via western blot. (**B**) The quantification of osteogenic-related protein, respectively. Data presented as mean ± SEM, *n* = 3 for each group. “*” indicates a significant difference (*p* < 0.05) when compared to CS scaffold. “Ctl” represented cells that grew in empty wells without any scaffolds.

Western blot technique was used to detect for the level of protein expression in Ctl, CS, and SrCS (Figure 10). ALP expression was measured as an early marker of maintenance of osteoblastic phenotype and BSP expression was measured as an assessment of bone formation as BSP is a critical component of the bone extracellular matrix [46]. ALP is mainly involved in the initial processes of bone extracellular matrix mineralization. OC, on the other hand, is a non-collagenous protein hormone that is secreted only by osteoblast and is involved in the process of bone formation. OC has been used as a serum marker for osteoblastic bone formation for a long time but it was only until recently that new genetic and pharmacological evidence pointed to a newly discovered hormonal role for OC. All the indicated markers served to assess the level of differentiation and bone tissue secretion to determine for the functionality of SrCS scaffolds. From Figure 10A, it is clearly seen that SrCS induces higher expression of ALP, BSP, and OC as compared to CS and Ctl (*p* < 0.05). Higher ALP expression indicated that there was quicker mineralization on the SrCS scaffold as compared to the rest. It was hypothesized that improved hydrophilicity led to enhanced adhesion, proliferation and thus higher amount of bone-related proteins secreted [47]. On the other hand, another hypothesis made by others states that when cells are in contact with rougher surfaces, their cytoskeleton would reorganize via rearrangement of actin filaments which led to a different gene expression profile [48]. Both hypotheses can be correlated and confirmed with the above-mentioned data. From Figure 10B, quantitative analysis showed that there was significantly higher expression of ALP, BSP, and OC from SrCS as compared to CS and Ctl. Therefore, it was further demonstrated that SrCS scaffolds were capable of inducing cellular differentiation and bone tissue secretion.

### 2.7. MSCs Secretion of Factors Affecting Osteoclast Formation on SrCS or CS Scaffold

**Figure 11:**
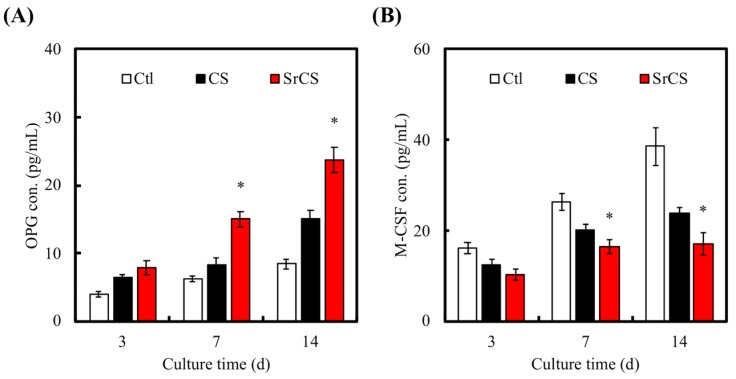
Proteins of (**A**) OPG and (**B**) M-CSF secretion by MSCs were measured by enzyme-linked immunosorbent assay. Data presented as mean ± SEM, *n* = 6 for each group. “*” indicates a significant difference (*p* < 0.05) when compared to CS scaffold. “Ctl” represented cells that grew in empty wells without any scaffolds.

In this study, OPG and M-CSF expression was measured using ELISA and result was shown in Figure 11. As seen from Figure 11, cells cultured on both CS and SrCS scaffolds showed elevated OPG secretions and decreased M-CSF secretions as compared to Ctl. However, there were no significant differences of both OPG and M-CSF secretion between CS and Ctl. On the other hand, SrCS had significant differences between these three groups after 7 days of culture. Of which, SrCS had 23.73 pg/mL of OPG after 14 days of culture as compared to 15.04 pg/mL for the CS group and 8.43 pg/mL for Ctl. This translates to approximately an 300% and 150% increment of OPG secretion as compared to CS and Ctl, respectively. In addition, after 7 days of culture, SrCS had 15.02 pg/mL of OPG secretion as compared to 8.26 pg/mL and 6.24 pg/mL for the CS and Ctl group, respectively. The concentration of M-CSF in the corresponding scaffolds at days 3 and 7 were as follows: day 3 (Ctl: 26.40 pg/mL, CS: 20.27 pg/mL and SrCS: 16.50 pg/mL) and day 7 (Ctl: 38.60 pg/mL, CS: 23.78 pg/mL and SrCS: 17.05 pg/mL). OPG is a critical protein in regulating bone homeostasis by maintaining balance between bone formation by osteoblasts and bone resorption by osteoclasts [49]. In addition, OPG is involved in inhibiting a pathway known as the RANK/RANKL pathway whereby activation of such pathways would lead to break down of bones. In short, osteoblasts secrete RANKL which would bind to RANK receptors found on osteoclast progenitor cells. After which, various downstream activation of transcription factors and gene expressions would eventually lead to differentiation of mature osteoclasts from the progenitor cells [50]. Mature osteoclasts would then bind to bone and secretion digestive enzymes to break down the bone. In this case, OPG acts as a decoy receptor for RANKL and thus inhibit bone resorption by not having downstream second messengers and activation factors. Similarly, osteoblasts can also secrete M-CSF which act in a paracrine fashion to activate osteoclast and thus leading to bone resorption. As it is a hematopoietic growth factor, M-CSF was known to bind to CSF1R receptors on osteoclast progenitor cells and upregulate growth and differentiation via the RANK pathway [51]. In short, bone homeostasis is a complex process with many signaling molecules involved, of which OPG and M-CSF are part of. From this prior result, SrCS modification was able to enhance OPG secretion and downregulate M-CSF and thus it was hypothesized that bone homeostasis was disrupted and tilted which could probably lead to enhanced bone formation over bone resorption.

### 2.8. Mineralization

**Figure 12:**
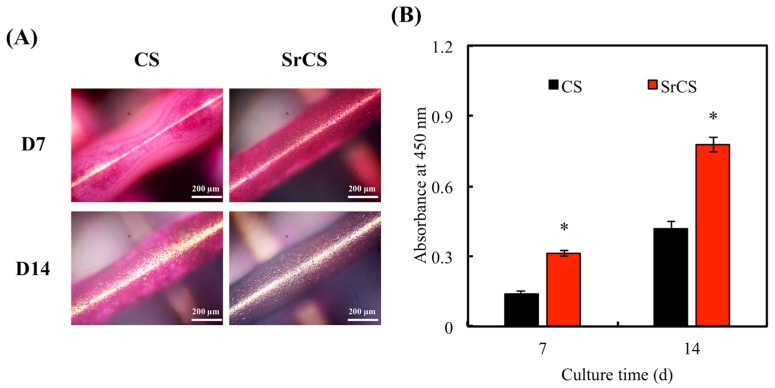
(**A**) Alizarin Red S staining and (**B**) quantification of calcium mineral deposits by MSCs. “*” indicates a significant difference (*p* < 0.05) when compared to CS scaffold. Data presented as mean ± SEM, *n* = 3 for each group.

In addition, Alizarin Red S staining and calcium quantification was also used to evaluate for calcium depositions (Figure 12A). It can be clearly seen that SrCS scaffolds had a higher amount of calcium mineral deposits as indicated by the strong pink staining. On day 14, the alizarin red S staining displayed a dark pinkish-purple outlook thus indicating a high amount of calcium deposits. Similarly, calcium quantification results showed that calcium depositions were significantly higher in the SrCS group as compared to the others from day 7 onwards (Figure 12B). These results were in good agreement with the above results by suggesting that higher proliferation, enhanced cellular adhesion and increased MAPK expression could play a role in subsequent increased calcium deposition. Furthermore, it was also reported that Sr had an additive effect on proliferation and differentiation of MSCs and that Sr promoted differentiation of MSCs to osteogenic lineage as compared with neat calcium materials [52].

### 2.9. In Vivo Bone Regeneration

**Figure 13:**
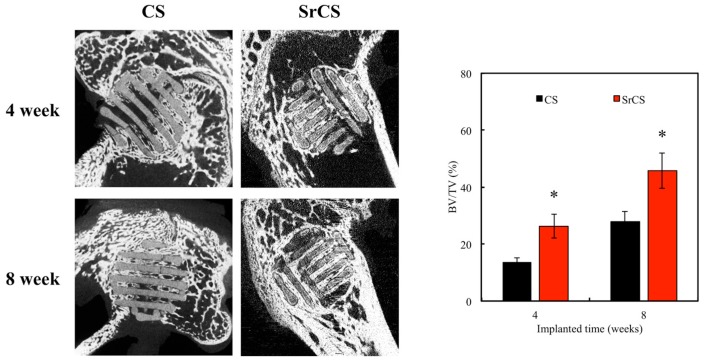
The µ-CT image showing the morphology of bone growth at fixed sized critical lesion after undergoing 4- and 8-weeks of regeneration with CS and SrCS scaffolds. Data analysis of relative bone mass volume (BV/TV) at fixed sized critical lesion after regeneration. “*” indicates a significant difference (*p* < 0.05) when compared to CS.

The in vivo regenerative efficacy of CS and SrCS scaffolds for bone defect was further investigated in rabbit femur defect model. To further testify the effect of Sr-contained on bone regeneration, in vivo assays were performed on white New Zealand rabbits. The results demonstrated that SrCS scaffold had greater bone regeneration in the femur defects both at 4- and 8-weeks post implantation compared with CS scaffolds. The µCT images (Figure 13) indicated significantly more new bone formation in the SrCS compared with the CS group. The BV/TV ratio of the SrCS scaffold (26.3 ± 1.9% and 45.7 ± 6.2%) was significantly higher compared with the CS scaffold (13.4 ± 1.6% and 27.9 ± 3.6%) at weeks 4 and 8, respectively (*p* < 0.05).

**Figure 14:**
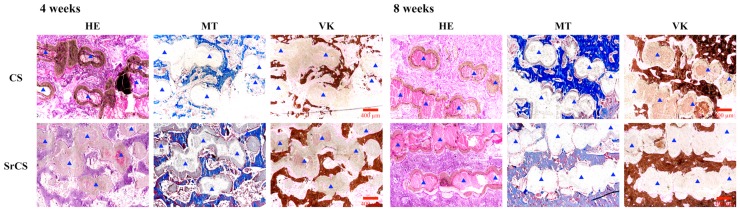
Histological analysis of new bone regeneration around and within the scaffolds in the rabbit femoral defect model. Left: hematoxylin and eosin (HE) stain; Middle: Masson’s trichrome (MT) stain; Right: Von Kossa (VK) stain of regenerated bone mass after 4- and 8-weeks of regeneration of in vivo experiment. The blue triangle indicated the scaffolds. The scale bar is 400 µm.

Figure 14 showed the histological evaluation results of the CS and SrCS groups at 4 and 8 weeks post implantation. In vivo tests showed increased bone tissue formation in the SrCS group at both 4 and 8 weeks post implantation. Masson trichrome stains mature bone tissues and newly regenerated bones in different shades of bright red and blue respectively. After a month of implantation, it can be seen that there was new bone formation, blue staining, in between the scaffold thus indicating that there was the presence of new bone tissue growth into the tissue. The blue stain was totally encapsulated by the scaffold, thus indicating that the bones were growing into the degrading scaffold. After 4 weeks, there was a darker shade of blue from the SrCS group as compared to CS, thus indicating increased amount of new bone formation. However, it was not so for week 8. Similarly, VK staining showed increase amount of bone tissue regeneration in SrCS group as compared to the CS group. Extracellular mineralization was obvious from the brown staining in the VK stain. Moreover, the quantitative analysis of the new bone area at 4- and 8-weeks was calculated from the corresponding VK staining images. The amount of new bone regeneration in the SrCS scaffold (51.1 ± 4.0%) was higher than that in the CS scaffold (25.6 ± 4.3%) after 4 weeks. The results demonstrated that the SrCS scaffold possessed excellent bone regeneration ability and high efficiency of bone formation. Generally, both CS and SrCS scaffolds induce bone tissue regeneration but it can be clearly indicated from Figure 14 that SrCS induces a higher amount of bone tissue regeneration as compared to CS due to the increased shades of staining in the different stains below. Furthermore, several studies had demonstrated that Sr ion has dual roles in bone metabolism by stimulating bone regeneration and inhibiting bone resorption [53]. The main mechanism is thought to lie in Sr ion possessing the ability not only to enhance the osteogenesis differentiation of MSCs via phosphating the Ras/MAPK signaling pathway but also to suppress the osteoclastogenic differentiation by inhibiting RANKL expression in MSCs [54]. Investigators had also indicated that Sr-contained HA cement and scaffolds could promote early angiogenesis after implantation into the critical size of bone defect and significantly enhance new bone regeneration [55,56].

## 3. Materials and Methods

### 3.1. Synthesis of Sr-Contained CS Powder and Preparation of Scaffolds

CS-based bioceramic were synthesized using methods established by previous report [57]. Analytically graded 70% calcium oxide (CaO, Sigma-Aldrich, St. Louis, MO, USA), 25% silicon dioxide (SiO_2_, Sigma-Aldrich), and 5% alumina oxide (Al_2_O_3_, Sigma-Aldrich) were mixed and sintered. To synthesize SrCS, 20% strontium oxide (SrO, Sigma-Aldrich) was used as the doping agent to replace CaO. The mixture oxide was then sintered at 1400 °C or 2 h and allowed to cool to room temperature before wet grinding with agate milling balls and 99.5% ethanol in a planetary ball mill (Retsch PM-100, Retsch GmbH, Germany) for 6 h. After which, the mixture was placed to dry in an oven for 12 h.

Prior to printing of scaffolds, first, CS powder was first stirred in 99.5% ethanol at a concentration of 0.1 g/mL. Polycaprolactone (PCL, Mw = 43,000–50,000, Polysciences, Warrington, PA, USA) was melted at 200 °C on a hotplate. After which, CS-ethanol solutions were then added to melted PCL and stirred until the ethanol was fully evaporated. Sr-doped powder was treated in a similar manner to CS. Next, the prepared injectable mixture was transferred to a cartridge and fixed on the BioScaffolder for fabrication of scaffolds (BioScaffolder 3.1, GeSiM, Großerkmannsdorf, Germany). The printing model was designed by a built-in software and determined to have a strut and line width of 500 μm. The scaffold was printed with a needle size of 400 μm, printing pressure of 200–300 kPa, and printing speed of 1.5–2 mm/s. The two group of specimens with and without Sr-dopants were coded as SrCS and CS, respectively.

### 3.2. Characterization of Physiochemical Properties of Scaffolds

To determine for the hydrophilicity of the composite, distilled water was dripped onto the surface of the scaffold at room temperature and the images were photographed using an USB digital microscope (Jiusion, Shenzhen, China). Images were taken once the water was dripped onto the scaffold and the static contact angle the images were analyzed and processed using ImageJ (National Institutes of Health, Bethesda, MD, USA). Three different sets were done for each scaffold and the result was averaged. In addition, X-ray diffractometry (XRD; Bruker D8 SSS, Karlsruhe, Germany) was used to characterize for the composition of the composite. The diffraction pattern was obtained on 2θ ranging from 20° to 50° with scanning steps of 1°/min. Furthermore, a scanning electron microscopy (FE-SEM; JEOL JSM-6700F, Tokyo, Japan) was used to take images of the scaffold to allow observation of the microstructural surface of the scaffolds. Briefly, specimens were mounted on the copper holder with conductive tape and sprayed with a nano-scale thickness layer of gold. Images were taken at a lower secondary electron image (LEI) mode and a 3 kV acceleration voltage. In addition, an EZ-Test machine (Shimadzu, Kyoto, Japan) was used to obtain for the stress–strain profiles of the scaffold. In short, a compressive force perpendicular to the shaft was applied to the top of the scaffold (6.5 × 6.5 × 10 mm) at a compression speed of 1 mm/min. The experiment was done with six specimens in each group.

### 3.3. In Vitro Biological Activity

The two sets of scaffolds were first placed into absolute ethanol for ultrasonic cleaning and subsequently left to dry before being placed into a centrifuge tube containing simulated body fluid (SBF). The SBF solution has similar ionic composition to human blood plasma and consists of 7.9949 g of NaCl, 0.3528 g of NaHCO_3_, 0.2235 g of KCl, 0.147 g of K_2_HPO_4_, 0.305 g of MgCl_2_·6H_2_O, 0.2775 g of CaCl_2_, and 0.071 of g Na_2_SO_4_ in 1000 mL of distilled H_2_O. Tubes were then sealed and placed in an oven at 37 °C for predetermined durations. Scaffolds were then taken out, gently washed thrice with deionized water and absolute ethanol and then dried in an oven at 40 °C. Thereafter, the deposition of hydroxyapatite on the surface of the scaffold was observed using SEM and the ions released were tested using an inductively coupled plasma-atomic emission spectrometer (ICP-AES; Perkin-Elmer OPT 1MA 3000DV, Shelton, CT, USA). The variation of weight loss in scaffolds and pH value of the residual SBF were also documented. The test was conducted thrice with the result averaged.

### 3.4. Biocompatibility of Scaffolds Extract Media

Quantitative analysis of cytotoxicity was done using mouse fibroblasts L929 cell line and performed in accordance to the revised edition of ISO 10993-12. Briefly, we printed CS and SrCS scaffolds which were subsequently washed with phosphate buffered saline (PBS, Invitrogen, Carlsbad, CA, USA) three times, followed by sterilization in 75% ethanol at room temperature for 30 min. The sterilized CS or SrCS scaffolds were first placed in a cell culture plate containing fresh medium in order to obtain scaffold extracts. The standard culture conditions were as follows: standard conditions of 1 g/10 mL and 37 °C for 24 h. Then, L929 cells (10^5^ cells) were seeded in a 6-cm dish with Dulbecco’s modified Eagle’s medium (DMEM, Invitrogen) at 37 °C for 24 h. Following which, DMEM was removed and replaced with the scaffold extract. After 1 day, the cells in the medium were first collected after cultivation. On the other hand, the cells attached on the culture plate were dissociated from adherent surfaces by dispensing 0.5% trypsin-EDTA covered the monolayer of cells. Immediately after, both cells in the medium and trypsinized cells were together centrifuged and removed the solution. Subsequently, PBS were then added to resuspend cells. In order to intercalate dye using acridine orange/propidium iodide (AO/PI) (Logos Biosystems, Gyeonggi-do, South Korea) to permeate both live and dead cells, 2 μL AO/PI and 18 μL cell sample were mixed and counted. The results were analyzed using LUNA-FLTM Automated Fluorescence Cell Counter (Logos Biosystems). The cells cultured on the cell culture plate without extracts were served as a control group.

### 3.5. Cell Proliferation and Morphology Observation on SrCS Scaffolds

The human mesenchymal stem cells (MSCs) used in this study were obtained from the Bioresource Collection and Research Center (BCRC, Hsin-Chu, Taiwan). The cells were cultivated in mesenchymal stem cell medium (#7501, Sciencell, Carlsbad, CA, USA) to passage 3–6 in an incubator with an atmosphere of 37 °C and 5% CO_2_/95% air. Prior to cell culture on the SrCS, the scaffolds were immersed in 75% ethanol and sterilized for 30 min under UV light in the laminar flow. The cell suspension (5 × 10^4^ cells/500 µL/scaffold) was seeded on different scaffolds for cell proliferation assay (*n* = 3) using PrestoBlue™ assay (Invitrogen) after 1, 3, and 7 days in culture. Briefly, 30 μL of PrestoBlue solution and 270 μL of DMEM were mixed and added to the culture well, and left to react in an incubator for 30 min. Then, 100 μL of the solution in each well was pipetted from the culture well and transferred to a fresh 96-well microplate. The absorbance of each well was determined using Tecan Infinite 200^®^ PRO microplate reader (Tecan, Männedorf, Switzerland) at an excitation wavelength of 570 nm and a reference wavelength of 600 nm. The cells directly cultured on the cell culture plate (without scaffold) were used as a control group (Ctl). In addition, cells were washed with PBS and fixed in 4% paraformaldehyde (Sigma-Aldrich) solution for 20 min at room temperature. Next, cells were permeabilized in PBS containing 0.1% Triton X-100 (Sigma-Aldrich). F-actin cytoskeleton was stained with fluorescent dye Alexa Fluor 488 (Invitrogen) conjugated to phalloidin to observe for morphology and cellular distribution. After washing with PBS, the morphology of the cells was observed using Leica TCS SP8 X white light laser confocal microscope (Leica Microsystems GmbH, Wetzlar, Hessen, Germany).

### 3.6. Western Blotting

In this assay, the 12 h and 24 h of culture duration was predetermined for the MAPK pathway assay and 7 days of culture duration was predetermined for the osteogenic-related protein. Firstly, cells were washed thrice with PBS and lysed with NP40 (Thermo Fisher, Waltham, MA, USA). The total protein concentrations were measuring using Quick StartTM Bradford protein assay kit (Bio-Rad, Hercules, CA, USA). Sample buffer was added and the solution was placed in a dry bath at 95 °C for 10 min to allow denaturation of the protein. Cell lysates of 40 μg protein/sample were segregated using the sodium dodecyl sulfate-polyacrylamide (SDS)-polyacrylamide gel electrophoresis (SDS-PAGE) that was prepared according to the kDa of the target protein antibody. After the end of the electrophoresis, the protein was transferred to a PVDF membrane and blocked with 5% BSA for 1 h. Antibody concentration was recommended by referring to the datasheet to add the diluted primary antibody—ERK, pERK, p38, pp38, alkaline phosphatase (ALP), bone sialoprotein (BSP), and osteocalcin (OC) from GeneTex (San Antonio, TX, USA). It was then placed on a 4 °C shaker and incubated overnight. The PVDF membrane was washed 3 times with TBST for 5 to 10 min each, then the secondary antibody was added and incubated for 1 h at room temperature. Protein bands were visualized by Vilber FUSION SOLO image scanning to detect the protein expression. Using β-actin (GeneTex) as an internal reference protein, the test was repeated in triplicate.

### 3.7. Feed-Back Regulation to Osteoclastogenesis by MSCs on Scaffold Surfaces

MSCs were seeded onto 3D-printed CS or SrCS scaffolds and cultured for 3, 7, and 14 days as described above. After each predetermined duration, the medium was centrifuged and stored frozen at −80 °C until further quantification. The concentrations of osteoprotegerin (OPG) and macrophage colony-stimulating factor (M-CSF) were secreted from the cells analyzed using ELISA kit (Abcam, Cambridge, MA, USA) according to the manufacturer’s instructions.

### 3.8. Mineralization

After cultured for 14 days, formation of mineralized bone nodules were observed by staining of the accumulated calcium with alizarin red S staining. The level of mineralization was determined after the cells were cultured on scaffolds with an osteogenic medium (StemPro™ osteogenesis differentiation kit, Invitrogen). Briefly, the sample was placed in 4% paraformaldehyde (Sigma-Aldrich) for 15 min, followed by the addition of 0.5%, pH 4.0 alizarin red S (Sigma-Aldrich) staining and incubated for 15 min at room temperature. The cells were then washed with PBS solution before having the calcium-chelated alizarin stain with 20% methanol and 10% acetic acid. After 15 min, the liquid was transferred to a 96-well plate and the alizarin red was quantified using Tecan Infinite 200^®^ PRO microplate reader at 450 nm.

### 3.9. Femoral Critical-Sized Bone Defect Rabbit Model

All in vivo experimental protocols and statements confirm that all of the methods were carried out in accordance with relevant guidelines and regulations. The statement to confirm that all experimental protocols were approved by the Ethical Committee for Animal Experiments of China Medical University, Taichung, Taiwan (CMUIACUC-2018-090, 2017/12). A critical-sized bone defect in the distal femoral epiphysis with a diameter of 6 mm and a depth of 6 mm was done on New Zealand male rabbits (mean weight: 1.8 kg). The experimental procedure was as follows: firstly, the white rabbit was anesthetized with injectable chlorohexidine, and then continuously anesthetized with 5% isoflurane in 100% oxygen using a gas anesthesia machine (Engler ADS1000). Subsequently, the hair of the hind legs was shaved with an electrical shaver and further disinfected with alcohol and iodine. A scalpel was used to dissect the skin and the dissection stretches from the outside of the thigh to the inside of calf. The muscle fascia was then dissected until the femur was exposed. Care was taken during the procedure to avoid dissecting too much muscle and essential structures such as nerves and blood vessels. A dental handpiece was used to create a defect and the broken bones were removed using saline and surgical probe. The scaffold was then implanted at the defect site, followed by subsequent wound closure and application of anti-inflammatory ointment onto the suture site.

### 3.10. In Vivo Newly Formed Bone and Histological Analysis

The animals were sacrificed after four and eight weeks with proper approved procedures. Animals were properly anesthetized before sacrificing with carbon dioxide. Carbon dioxide was continuously supplied for five minutes. After which, the animals were observed for another two minutes to ensure that there was no other signs of life and vital signs. The scaffolds and control were removed from the femur and micro-CT scanning and histological analysis were performed. Reconstruction images of the femoral defect were obtained using a µCT (SkyScan 1076, SkyScan Inc., Kontich, Belgium) equipped with a 1.4 M CCD camera. For newly formed bone tissues, reconstructed data were analyzed by Avizo 8.1 (Visualization Sciences Group, FEI, Hillsboro, OR, USA) as well for determining the bone volume per tissue volume (BV/TV). In addition, for histological analysis, the specimens were first fixed in 10% formalin for 48 h. Thereafter, the sample was washed with PBS, without decalcified, and embedded in OCT (KMA-0100-00A, CellPath Ltd., Newtown, Wales, UK). Then, the 6 µm sections were cut on a Leica CM3050S cryostat (Leica Microsystems, Wetzlar, Germany) and stained using hematoxylin-eosin (H&E), Masson’s Trichrome (MT, ScyTek Lab., West Logan, UT, USA) and Von Kossa (VK, ScyTek) according to manufacturer’s protocols. Finally, observation was carried out through Zeiss Axioskop2 microscope. In addition, the new bone area (VK staining) was visualized using ImageJ to assess for the proportion of new bone and defect area.

### 3.11. Statistical Analysis

One-way variance statistical analysis was used to assess significant differences in each group, and Scheffe’s multiple comparison test was used for each specimen. *P* < 0.05 was considered statistically significant.

## 4. Conclusions

In this study, SrCS powders were successfully synthesized with SrCS scaffolds successfully manufactured using 3D printing technique. The compressive strength of SrCS scaffolds were shown to be 2-times higher than CS scaffolds. In addition, SrCS scaffolds demonstrated good apatite-forming bioactivity with sustained release of Si and Sr ions. The in vitro tests showed that SrCS scaffolds possessed excellent biocompatibility which led to enhanced adhesion, proliferation, and differentiation of MSCs. In addition, the SrCS scaffolds were able to enhance MSCs synthesis of osteoprotegerin (OPG) and suppress macrophage colony-stimulating factor (M-CSF) thus disrupting normal bone homeostasis and leading to enhanced bone formation over bone resorption. In addition, the implanted SrCS scaffolds were found to be able to promote new blood vessel growth and new bone regeneration in in vivo tests. Therefore, we hypothesized that these 3D-printed SrCS scaffolds may be a promising and potential candidate for bone defect repair in the future.

## References

[1] Rodriguez-Palomo A., Monopoli D., Afonso H., Izquierdo-Barba I., Vallet-Regí M. (2016). Surface zwitterionization of customized 3D Ti6Al4V scaffolds: A promising alternative to eradicate bone infection. J. Mater. Chem. B.

[2] Shim J.H., Won J.Y., Park J.H., Bae J.H., Ahn G., Kim C.H., Lim D.H., Cho D.W., Yun W.S., Bae E.B. (2017). Effects of 3D-printed polycaprolactone/β-tricalcium phosphate membranes on guided bone regeneration. Int. J. Mol. Sci..

[3] Luo Y., Li Y., Qin X., Wa Q. (2018). 3D printing of concentrated alginate/gelatin scaffolds with homogeneous nano apatite coating for bone tissue engineering. Mater. Des..

[4] Deng Y., Jiang C., Li C., Li T., Peng M., Wang J., Dai K. (2017). 3D printed scaffolds of calcium silicate-doped β-TCP synergize with co-cultured endothelial and stromal cells to promote vascularization and bone formation. Sci. Rep..

[5] Gómez-Lizárraga K.K., Flores-Morales C., Del Prado-Audelo M.L., Álvarez-Pérez M.A., Piña-Barba M.C., Escobedo C. (2017). Polycaprolactone- and polycaprolactone/ceramic-based 3D-bioplotted porous scaffolds for bone regeneration: A comparative study. Mater. Sci. Eng. C Mater. Biol. Appl..

[6] Park S., Lee H.J., Kim K.S., Lee S., Lee J.T., Kim S.Y., Chang N.H., Park S.Y. (2018). In vivo evaluation of 3D-printed polycaprolactone scaffold implantation combined with β-TCP powder for alveolar bone augmentation in a beagle defect model. Materials.

[7] Lee H., Yang G.H., Kim M., Lee J., Huh J., Kim G. (2018). Fabrication of micro/nanoporous collagen/dECM/silk-fibroin biocomposite scaffolds using a low temperature 3D printing process for bone tissue regeneration. Mater. Sci. Eng. C Mater. Biol. Appl..

[8] Wang S., Li R., Li D., Zhang Z.-Y., Liu G., Liang H., Qin Y., Yu J., Li Y. (2018). Fabrication of bioactive 3D printed porous titanium implants with Sr ion-incorporated zeolite coatings for bone ingrowth. J. Mater. Chem. B.

[9] Shao H., Ke X., Liu A., Sun M., He Y., Yang X., Fu J., Liu Y., Zhang L., Yang G. (2017). Bone regeneration in 3D printing bioactive ceramic scaffolds with improved tissue/material interface pore architecture in thin-wall bone defect. Biofabrication.

[10] Anada T., Pan C.C., Stahl A.M., Mori S., Fukuda J., Suzuki O., Yang Y. (2019). Vascularized bone-mimetic hydrogel constructs by 3D bioprinting to promote osteogenesis and angiogenesis. Int. J. Mol. Sci..

[11] Chen Y.W., Hsu T.T., Wang K., Shie M.Y. (2016). Preparation of the fast setting and degrading Ca-Si-Mg cement with both odontogenesis and angiogenesis differentiation of human periodontal ligament cells. Mater. Sci. Eng. C Mater. Biol. Appl..

[12] Wu C., Chang J., Zhai W., Ni S. (2007). A novel bioactive porous bredigite (Ca7MgSi4O16) scaffold with biomimetic apatite layer for bone tissue engineering. J. Mater. Sci. Mater. Med..

[13] Pan C., Chen L., Wu R., Shan H., Zhou Z., Lin Y., Yu X., Yan L., Wu C. (2018). Lithium-containing biomaterials inhibit osteoclastogenesis of macrophages in vitro and osteolysis in vivo. J. Mater. Chem. B.

[14] Xiao D., Yang F., Zhou X., Chen Z., Duan K., Weng J., Feng G. (2017). Small organic molecule-mediated hydrothermal synthesis of hierarchical porous hydroxyapatite microspheres by the incorporation of copper ions. RSC Adv..

[15] Hao F., Qin L., Liu J., Chang J., Huan Z., Wu L. (2018). Assessment of calcium sulfate hemihydrate–tricalcium silicate composite for bone healing in a rabbit femoral condyle model. Mater. Sci. Eng. C Mater. Biol. Appl..

[16] Shie M.Y., Ding S.J., Chang H.C. (2011). The role of silicon in osteoblast-like cell proliferation and apoptosis. Acta Biomater..

[17] Ding Z., Li H., Wei J., Li R., Yan Y. (2018). Developing a novel magnesium glycerophosphate/silicate-based organic-inorganic composite cement for bone repair. Mater. Sci. Eng. C Mater. Biol. Appl..

[18] Tsai C.H., Hung C.H., Kuo C.N., Chen C.Y., Peng Y.N., Shie M.Y. (2019). Improved bioactivity of 3D printed porous titanium alloy scaffold with chitosan/magnesium-calcium silicate composite for orthopaedic applications. Materials.

[19] Huang K.H., Chen Y.W., Wang C.Y., Lin Y.H., Wu Y.H., Shie M.Y., Lin C.P. (2018). Enhanced capability of BMP-2-loaded mesoporous calcium silicate scaffolds to induce odontogenic differentiation of human dental pulp cells. J. Endod..

[20] Chen Y.W., Shen Y.F., Ho C.C., Yu J., Wu Y.H., Wang K., Shih C.T., Shie M.Y. (2018). Osteogenic and angiogenic potentials of the cell-laden hydrogel/mussel-inspired calcium silicate complex hierarchical porous scaffold fabricated by 3D bioprinting. Mater. Sci. Eng. C Mater. Biol. Appl..

[21] Sriranganathan D., Kanwal N., Hing K.A., Hill R.G. (2016). Strontium substituted bioactive glasses for tissue engineered scaffolds: The importance of octacalcium phosphate. J. Mater. Sci. Mater. Med..

[22] Schumacher M., Gelinsky M. (2015). Strontium modified calcium phosphate cements—Approaches towards targeted stimulation of bone turnover. J. Mater. Chem. B.

[23] Yang F., Yang D., Tu J., Zheng Q., Cai L., Wang L. (2011). Strontium enhances osteogenic differentiation of mesenchymal stem cells and in vivo bone formation by activating Wnt/catenin signaling. Stem Cells.

[24] Lin K., Xia L., Li H., Jiang X., Pan H., Xu Y., Lu W.W., Zhang Z., Chang J. (2013). Enhanced osteoporotic bone regeneration by strontium-substituted calcium silicate bioactive ceramics. Biomaterials.

[25] Hoppe A., Güldal N.S., Boccaccini A.R. (2011). A review of the biological response to ionic dissolution products from bioactive glasses and glass-ceramics. Biomaterials.

[26] Quade M., Schumacher M., Bernhardt A., Lode A., Kampschulte M., Voß A., Simon P., Uckermann O., Kirsch M., Gelinsky M. (2018). Strontium-modification of porous scaffolds from mineralized collagen for potential use in bone defect therapy. Mater. Sci. Eng. C Mater. Biol. Appl..

[27] No Y.J., Roohaniesfahani S., Lu Z., Shi J., Zreiqat H. (2017). Strontium-doped calcium silicate bioceramic with enhanced in vitro osteogenic properties. Biomed. Mater..

[28] Yang C., Huan Z., Wang X., Wu C., Chang J. (2018). 3D printed Fe scaffolds with HA nanocoating for bone regeneration. ACS Biomater. Sci. Eng..

[29] Basu S., Ghosh A., Barui A., Basu B. (2018). (Fe/Sr) codoped biphasic calcium phosphate with tailored osteoblast cell functionality. ACS Biomater. Sci. Eng..

[30] Lin Y.H., Chiu Y.C., Shen Y.F., Wu Y.H., Shie M.Y. (2018). Bioactive calcium silicate/poly-ε-caprolactone composite scaffolds 3D printed under mild conditions for bone tissue engineering. J. Mater. Sci. Mater. Med..

[31] Chiu Y.C., Fang H.Y., Hsu T.T., Lin C.Y., Shie M.Y. (2017). The characteristics of Mineral Trioxide Aggregate/polycaprolactone 3-dimensional scaffold with osteogenesis properties for tissue regeneration. J. Endod..

[32] Uswatta S.P., Okeke I.U., Jayasuriya A.C. (2016). Injectable porous nano-hydroxyapatite/chitosan/tripolyphosphate scaffolds with improved compressive strength for bone regeneration. Mater. Sci. Eng. C Mater. Biol. Appl..

[33] Lei Y., Xu Z., Ke Q., Yin W., Chen Y., Zhang C., Guo Y. (2017). Strontium hydroxyapatite/chitosan nanohybrid scaffolds with enhanced osteoinductivity for bone tissue engineering. Mater. Sci. Eng. C Mater. Biol. Appl..

[34] Mehrotra S., Moses J.C., Bandyopadhyay A., Mandal B.B. (2019). 3D printing/bioprinting based tailoring of in vitro tissue models: Recent advances and challenges. ACS Appl. Bio Mater..

[35] Huang Y., Wu C., Zhang X., Chang J., Dai K. (2018). Regulation of immune response by bioactive ions released from silicate bioceramics for bone regeneration. Acta Biomater..

[36] Zhu Y., Zhu M., He X., Zhang J., Tao C. (2013). Substitutions of strontium in mesoporous calcium silicate and their physicochemical and biological properties. Acta Biomater..

[37] Dow E.C., Stanbury J.B. (1960). Strontium and calcium metabolism in metabolic bone diseases. Am. Soc. Clin. Investig..

[38] Ke D., Tarafder S., Vahabzadeh S., Bose S. (2019). Effects of MgO, ZnO, SrO, and SiO_2_ in tricalcium phosphate scaffolds on in vitro gene expression and in vivo osteogenesis. Mater. Sci. Eng. C Mater. Biol. Appl..

[39] Lourenço A.H., Torres A.L., Vasconcelos D.P., Ribeiro-Machado C., Barbosa J.N., Barbosa M.A., Barrias C.C., Ribeiro C.C. (2019). Osteogenic, anti-osteoclastogenic and immunomodulatory properties of a strontium-releasing hybrid scaffold for bone repair. Mater. Sci. Eng. C Mater. Biol. Appl..

[40] Chen C.C., Yu J., Ng H.Y., Lee K.X., Chen C.C., Chen Y.S., Shie M.Y. (2018). The physicochemical properties of decellularized extracellular matrix-coated 3D printed poly(ε-caprolactone) nerve conduits for promoting Schwann cells proliferation and differentiation. Materials.

[41] Zhang W., Huang D., Zhao F., Gao W., Sun L., Li X., Chen X. (2018). Synergistic effect of strontium and silicon in strontium-substituted sub-micron bioactive glass for enhanced osteogenesis. Mater. Sci. Eng. C Mater. Biol. Appl..

[42] Wu C., Zhou Y.Z., Lin C., Chang J., Xiao Y. (2012). Strontium-containing mesoporous bioactive glass scaffolds with improved osteogenic/cementogenic differentiation of periodontal ligament cells for periodontal tissue engineering. Acta Biomater..

[43] Kendler D.L. (2006). Strontium ranelate—Data on vertebral and nonvertebral fracture efficacy and safety: Mechanism of action. Curr. Osteoporos. Rep..

[44] Shie M.Y., Ding S.J. (2013). Integrin binding and MAPK signal pathways in primary cell responses to surface chemistry of calcium silicate cements. Biomaterials.

[45] Ma H., Feng C., Chang J., Wu C. (2018). 3D-printed bioceramic scaffolds: From bone tissue engineering to tumor therapy. Acta Biomater..

[46] Zhu H., Zhai D., Lin C., Zhang Y., Huan Z., Chang J., Wu C. (2016). 3D plotting of highly uniform Sr_5_(PO_4_)_2_SiO_4_ bioceramic scaffolds for bone tissue engineering. J. Mater. Chem. B.

[47] Gong Z., Cheng H., Zhang M., Liu X., Zeng Y., Xiang K., Xu Y., Wang Y., Zhu Z. (2017). Osteogenic activity and angiogenesis of a SrTiO3 nano-gridding structure on titanium surface. J. Mater. Chem. B.

[48] Shie M.Y., Chang H.C., Ding S.J. (2014). Composition-dependent protein secretion and integrin level of osteoblastic cell on calcium silicate cements. J. Biomed. Mater. Res. Part A.

[49] Cheng Z., Landish B., Chi Z., Nannan C., Jingyu D., Sen L., Xiangjin L. (2018). 3D printing hydrogel with graphene oxide is functional in cartilage protection by influencing the signal pathway of Rank/Rankl/OPG. Mater. Sci. Eng. C Mater. Biol. Appl..

[50] Ma Q.L., Fang L., Jiang N., Zhang L., Wang Y., Zhang Y.M., Chen L.H. (2018). Bone mesenchymal stem cell secretion of sRANKL/OPG/M-CSF in response to macrophage-mediated inflammatory response influences osteogenesis on nanostructured Ti surfaces. Biomaterials.

[51] Boyle W.J., Simonet W.S., Lacey D.L. (2003). Osteoclast differentiation and activation. Nature.

[52] Xu K., Chen W., Mu C., Yu Y., Cai K. (2017). Strontium folic acid derivative functionalized titanium surfaces for enhanced osteogenic differentiation of mesenchymal stem cells in vitro and bone formation in vivo. J. Mater. Chem. B.

[53] Torres P.M.C., Marote A., Cerqueira A.R., Calado A.J., Abrantes J.C.C., Olhero S., da Cruz e Silva O.A.B., Vieira S.I., Ferreira J.M.F. (2017). Injectable MnSr-doped brushite bone cements with improved biological performance. J. Mater. Chem. B.

[54] Marie P.J., Felsenberg D., Brandi M.L. (2010). How strontium ranelate, via opposite effects on bone resorption and formation, prevents osteoporosis. Osteoporos. Int..

[55] Yan S., Xia P., Xu S., Zhang K., Li G., Cui L., Yin J. (2018). Nanocomposite porous microcarriers based on strontium-substituted HA-g-poly(γ-benzyl-L-glutamate) for bone tissue engineering. ACS Appl. Mater. Interfaces.

[56] Guo D., Xu K., Zhao X., Han Y. (2005). Development of a strontium-containing hydroxyapatite bone cement. Biomaterials.

[57] Wu Y.H., Chiu Y.C., Lin Y.H., Ho C.C., Shie M.Y., Chen Y.W. (2019). 3D-printed bioactive calcium silicate/poly-ε-caprolactone bioscaffolds modified with biomimetic extracellular matrices for bone regeneration. Int. J. Mol. Sci..

